# Inhibition of Glucose-Induced Glycation of HSA by Pioglitazone: Multi-Spectroscopic and Bioinformatic Evidence

**DOI:** 10.3390/molecules31091519

**Published:** 2026-05-03

**Authors:** Jihad Alrehaili, Razique Anwer

**Affiliations:** Department of Pathology, College of Medicine, Imam Mohammad Ibn Saud Islamic University (IMSIU), Riyadh 4233-13317, Saudi Arabia; jaalrehaili@imamu.edu.sa

**Keywords:** diabetes, advanced glycation end products (AGEs): pioglitazone, glycation, HSA, molecular dynamics simulations

## Abstract

Diabetes mellitus is a growing health concern that causes numerous complications. Glycation produces advanced glycation end-products (AGEs), which promote diabetic complications. Targeting glycation is a strategy for combating the progression of diabetic complications. Pioglitazone enhances insulin sensitivity in patients with type 2 diabetes mellitus, but its impact on glycation remains unclear. This study aims to evaluate whether pioglitazone can inhibit glucose-induced glycation of human serum albumin (HSA), using in vitro assays and in silico tools. Pioglitazone inhibited >70% of early glycation products and >75% of AGEs. The treatment also reduced free lysine modification and improved biochemical markers, including carbonyl and free thiol levels. Pioglitazone exhibited moderate binding affinity for HSA, with a binding constant of 10^4^ M^−1^. The interaction between pioglitazone and HSA was both spontaneous and entropically favourable. Molecular dynamics simulations revealed that the HSA–pioglitazone complex remained quite stable, with RMSF, RMSD, SASA, R_g_, and HSA’s secondary structure showing minimal changes throughout the simulation. The overall binding energy for HSA–pioglitazone complex formation was −30.06 ± 0.31 kcal mol^−1^, as obtained from MD simulations. The findings suggest that pioglitazone likely interacts with glycation-prone regions of HSA, as indicated by spectroscopic and docking analyses, and contributes to the reduction of glycation.

## 1. Introduction

Diabetes mellitus is a multifaceted metabolic condition marked by persistently elevated blood glucose levels. This prolonged hyperglycemia results from irregularities in the body’s ability to produce insulin, respond effectively to insulin, or both, leading to disturbances in glucose regulation. The increasing prevalence of diabetes poses significant public health challenges, as it is associated with numerous complications, like nephropathy, neuropathy, cardiovascular diseases, and retinopathy [1]. One of the critical biochemical processes known to contribute to the pathogenesis of these complications is glycation, a non-enzymatic reaction involving the free amino groups of proteins and reducing sugars. This reaction leads to the formation of AGEs, which are known to induce oxidative stress and inflammation and exacerbate diabetic complications [2].

Glycation occurs when excess glucose in the bloodstream reacts with proteins, leading to structural and functional modifications. This reaction can significantly alter the properties of proteins, including their biological activities and binding affinities [3]. For instance, glycated albumin, a major circulating protein, is not only a marker of glycaemic control but is also known to contribute to the onset of certain diabetic complications [1]. Elevated levels of glycated albumin are directly associated with increased risks of nephropathy and cardiovascular diseases in diabetic patients [4]. The accumulation of AGEs can lead to the cross-linking of proteins, impairing their function and promoting vascular damage, which is a hallmark of diabetes-related complications [5].

Targeting glycation presents a promising strategy for managing diabetes and reducing diabetic complications. Interventions aimed at reducing glycation or its effects could potentially mitigate the progression of diabetic complications. For example, pharmacological agents that inhibit AGE formation or enhance the clearance of glycated proteins may help restore normal physiological functions [2,6]. Additionally, lifestyle improvements, like dietary changes and regular physical activity, may improve glycaemic control and reduce AGE formation, thereby lowering the risk of complications [7]. Furthermore, emerging biomarkers like glycated albumin and fructosamine provide valuable insights into glycaemic status and can guide therapeutic decisions in diabetes management [8].

Pioglitazone is a thiazolidinedione-class medication primarily used to improve insulin sensitivity in patients with type 2 diabetes mellitus. Studies have reported that pioglitazone can modulate AGE-related biological processes beyond its glucose-lowering effects. Pioglitazone attenuates AGE-induced cellular dysfunction, apoptosis, and calcification in tendon-derived stem cells and reduces AGE-driven matrix changes in cartilage and other tissues, effects attributed to PPARγ-mediated anti-inflammatory, antioxidant, and autophagy-modulating actions rather than to the direct chemical trapping of reducing sugars [9]. Scientific investigations have highlighted its multifaceted mechanisms, particularly its interaction with mitochondrial proteins such as mitoNEET, an important protein involved in cellular energy metabolism. Binding studies have revealed that pioglitazone stabilizes mitoNEET, thereby enhancing its function and potentially mitigating oxidative stress associated with diabetes [10]. However, the precise effect of pioglitazone on glycation remains unclear. This study aimed to evaluate the effect of pioglitazone on glucose-induced glycation of HSA using spectroscopic and in silico approaches. It also aimed to determine whether direct binding to HSA could contribute to the observed antiglycation effect. HSA was selected because it is the most abundant plasma protein and a major carrier of endogenous and exogenous ligands. Glycation alters HSA’s conformation and ligand-binding sites, which can affect drug pharmacokinetics and reduce antioxidant capacity; elevated levels of glycated albumin are also associated with diabetic complications. Therefore, investigating whether pioglitazone can reduce HSA glycation is important both for understanding the potential off-target benefits of the drug and for assessing whether pioglitazone–HSA interactions might preserve albumin function under hyperglycemic conditions.

## 2. Results and Discussion

### 2.1. Pioglitazone Inhibits the Glycation of HSA

To examine the inhibitory effect of pioglitazone on HSA glycation, various important biochemical and biophysical assays were performed. The findings are discussed below.

#### 2.1.1. Examination of Fructosamine Content

**Figure 1:**
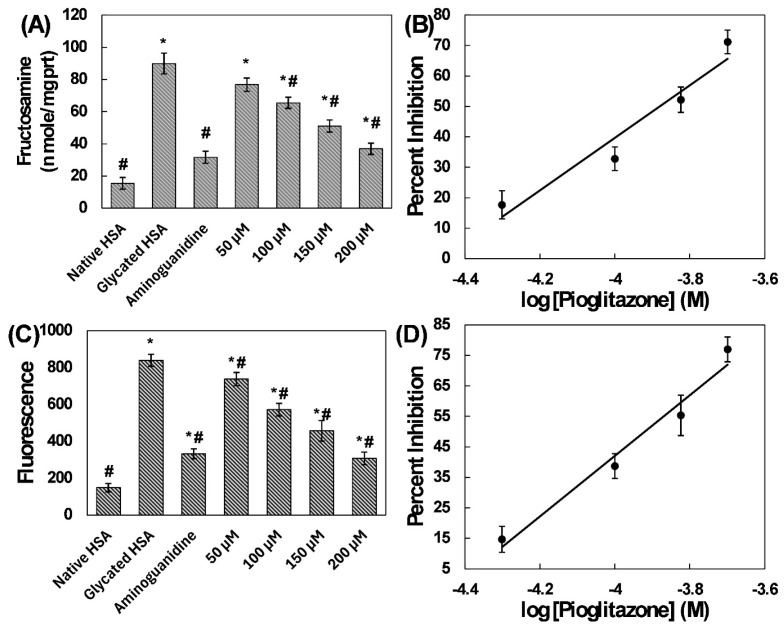
(**A**) Inhibition of fructosamine formation in HSA by pioglitazone. (**B**) IC_50_ plot for fructosamine inhibition. (**C**) Inhibition of fluorescent AGEs in HSA by varying concentrations of pioglitazone. (**D**) IC_50_ plot for fluorescent AGE inhibition. The emission signal was recorded at 440 nm following excitation at 370 nm. Aminoguanidine was used as a positive control. The fructosamine and fluorescence AGE assays were performed in quadruplicate, and the data is presented as mean values, with standard deviations indicated by error bars. # Indicates a statistically significant difference (*p* ≤ 0.05) compared to the glycated HSA group. * Indicates a statistically significant difference (*p* ≤ 0.05) compared to the native HSA group.

Glucose is the most abundant reducing sugar in blood and is commonly used in in vitro glycation models; other reducing sugars, such as fructose and ribose, are known to be more reactive and capable of producing AGEs [11]. Fructose has also been reported to generate AGEs, and ribose is frequently used in accelerated glycation assays because of its high reactivity [12]. We used a glucose-induced glycation model due to its physiological relevance. When reducing sugars react non-enzymatically with proteins, early glycation products are formed. Among these products, fructosamine is a well-known marker, and fructosamine estimation provides insights into the presence of early glycation products [13]. The protective role of pioglitazone against the formation of such glycation products was examined. Fructosamine levels were quantified, as depicted in Figure 1A. In the control HSA sample (native HSA), the fructosamine concentration was 15.42 ± 3.63 nmoles/mg protein. Glycation of HSA significantly enhanced the fructosamine level to 89.89 ± 6.48 nmoles/mg protein. Interestingly, the presence of pioglitazone reduced fructosamine formation in a dose-dependent manner. At the lowest concentration (50 µM), 17.66% inhibition of fructosamine formation was observed, which was statistically insignificant compared to glycated HSA. We observed 32.80% and 52.19% inhibition of fructosamine formation at pioglitazone concentrations of 100 and 150 µM, respectively. In the presence of 200 µM pioglitazone, more than 71% reduction in fructosamine content was observed. The IC_50_ for fructosamine inhibition by pioglitazone was 130.34 µM (Figure 1B). Aminoguanidine (positive control) inhibited fructosamine formation by 78.22%. Fructosamine levels serve as important markers in the development of diabetic complications. Glycation leads to the formation of Amadori products, which contribute to secondary complications in diabetes [14,15]. Previously, pioglitazone was found to inhibit the glycation of an eye lens protein α-crystallin [16]. Our findings highlight the potential therapeutic effect of pioglitazone in modulating fructosamine levels, which might prove beneficial in mitigating diabetic complications. Future studies should also evaluate pioglitazone using fructose- and ribose-induced glycation models, as well as in cellular and animal models, to further determine its protective effects.

#### 2.1.2. Examination of Fluorescent AGEs

AGEs are the final products of glycation, a process in which sugars react with proteins. AGEs comprise a diverse group of compounds that exhibit remarkable fluorescence when excited at a wavelength of 370 nm. Here, the protective effect of pioglitazone against the formation of fluorescent AGEs was investigated (Figure 1C). The control sample (native HSA) displayed lower fluorescence than the glycated HSA sample. The latter showed more than a fivefold increase in fluorescent AGEs compared to the native HSA sample. Interestingly, as the pioglitazone concentration increased, a progressive decrease in fluorescent AGEs was observed. Treatment with pioglitazone at 50 µM, 100 µM, and 150 µM resulted in 14.68%, 38.70%, and 55.33% reductions in fluorescent AGEs, respectively. A higher concentration (200 µM) resulted in a remarkable decrease of more than 75% in fluorescent AGEs. The IC_50_ for inhibition of fluorescent AGEs by pioglitazone was 119.94 µM (Figure 1D). Additionally, the positive control (aminoguanidine) exhibited an inhibitory effect of over 73.35% on AGE formation.

#### 2.1.3. Examination of Free Lysine Content

**Figure 2:**
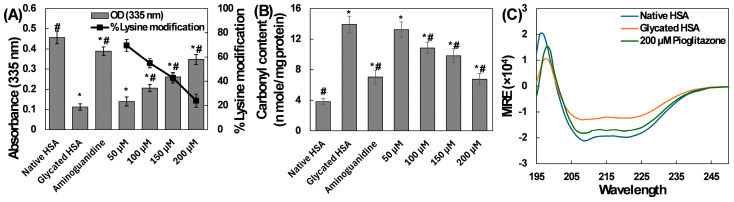
(**A**) Levels of lysine modification in native HSA, glycated HSA, and pioglitazone-treated HSA. (**B**) Carbonyl content in native HSA, glycated HSA, and pioglitazone-treated HSA. (**C**) Circular dichroism spectra of native HSA, glycated HSA, and 200 µM pioglitazone-treated HSA. Aminoguanidine was used as a positive control. The assays were performed in quadruplicate, and the data is presented as mean values, with standard deviations indicated by error bars. # Indicates a statistically significant difference (*p* ≤ 0.05) compared to the glycated HSA group. * Indicates a statistically significant difference (*p* ≤ 0.05) compared to the native HSA group.

Among the various amino acid residues susceptible to glycation, lysine is one of the most vulnerable targets [17]. Lysine has been reported to undergo glycation in several proteins, including serum albumin, hemoglobin, and crystallins. Besides lysine, arginine is another residue susceptible to glycation, followed by histidine and cysteine [18]. The glycation process involves the modification of free amino groups in lysine, leading to the formation of glycoxidation products like carboxyethyl lysine, vesper lysine, and carboxymethyl lysine [19]. Quantification of free lysine content in HSA samples is presented in Figure 2A, revealing that over 75% of free lysine was modified in the glycated HSA samples. In contrast, treatment with pioglitazone produced a dose-dependent effect on free lysine modification. Specifically, at pioglitazone concentrations of 50 µM, 100 µM, and 150 µM, the percentages of lysine modification were 69.56%, 54.89%, and 42.80%, respectively. Interestingly, at a higher concentration of 200 µM, <25% of free lysine showed modification. In the aminoguanidine-treated sample, the lysine modification was found to be 14.83%. These findings provide evidence for the protective role of pioglitazone against free lysine modification. One potential mechanism underlying this protective role is the binding of pioglitazone to free lysine groups, which may reduce glycation reactions. Notably, a similar observation was reported in an earlier study in which pioglitazone inhibited free lysine modification in α-crystallin [20].

#### 2.1.4. Determination of Carbonyl Content

In protein glycation, Schiff bases are important. These compounds initially form during the glycation process and subsequently give rise to stable ketoamines known as Amadori products. Via a series of chemical reactions, especially enediol reactions, protein glycation leads to the formation of carbonyl compounds [21]. The carbonyl levels in HSA samples are shown in Figure 2B. In native HSA, the carbonyl level was measured at 3.81 ± 0.40 nmoles/mg protein. However, in glycated HSA, this level increased significantly to 13.92 ± 1.09 nmoles/mg protein. To further explore this effect, the impact of pioglitazone on carbonyl levels was also examined. The presence of pioglitazone at concentrations of 50, 100, and 150 µM resulted in 6.74%, 30.37%, and 40.49% recovery of carbonyl content, respectively. Remarkably, at the higher concentration (200 µM), more than 70% recovery was observed. These findings suggest that pioglitazone either inhibits carbonyl formation or effectively scavenges free carbonyl species. Additionally, during glycation, superoxide radicals are generated, which subsequently transform into highly reactive hydroxyl radicals through the Fenton reaction. These hydroxyl radicals contribute to oxidative stress and cellular damage [22]. Glycation and subsequent glycoxidation generate reactive oxygen species and carbonyl derivatives that contribute to protein dysfunction and diabetic complications. The observed reduction in carbonyl content and the partial recovery of free thiols in pioglitazone-treated HSA suggest that the compound may mitigate glycoxidative damage in addition to reducing glycation adducts.

#### 2.1.5. Determination of α-Helix in HSA

The impact of pioglitazone on the secondary structure of HSA was investigated using CD spectroscopy. Inspection of the CD spectrum of the control has revealed a far-UV CD negative band in the 210–220 nm range (Figure 2C). The CD data was further analyzed to calculate MRE (molar ellipticity) and % α-helix using Equations (1) and (2) [23]:

(1) $$MRE=\frac{CD\left(mdeg\right)}{C_{p}nl\;\times 10}$$

(2) $$\%\alpha -helix=\frac{{(-MRE}_{208}-4000)}{33000-4000}\times 100$$

where *n* represents the number of HSA residues, *C_p_* is the HSA concentration, and *l* is the cuvette path length. The α-helix content of the HSA samples is detailed in Table 1. The native HSA sample exhibited 58.26% α-helix. Upon glycation, the α-helical content decreased to 31.04%. However, pioglitazone treatment (200 µM) restored the α-helical content to 49.02%. These findings support the idea that pioglitazone can protect the secondary structure of HSA during glycation.

Comparative analysis of the in vitro assays showed a consistent pattern supporting reduced HSA modification in the presence of pioglitazone. Fructosamine (early Amadori products) showed a modest decrease compared with glycated HSA. Fluorescent AGE measurements exhibited a larger and more consistent reduction, suggesting that the formation of late, fluorescent AGE species is more strongly inhibited. Free lysine modification assays showed reduced modification of ε-amino groups. Carbonyl content and free-thiol assays both indicated reduced glycoxidative damage (lower carbonyl levels and recovery of free thiols). CD spectra showed minimal changes in the global secondary structure, indicating that pioglitazone preserves the overall fold of HSA. Therefore, the various in vitro assays collectively support the conclusion that pioglitazone reduces HSA modification.

### 2.2. In Vitro Interaction of HSA and Pioglitazone

To further explore how pioglitazone may reduce HSA glycation, a series of in vitro studies, including steady-state fluorescence and site-marker analyses, was performed. The findings are discussed below.

#### 2.2.1. Fluorescence Quenching Assay

**Figure 3:**
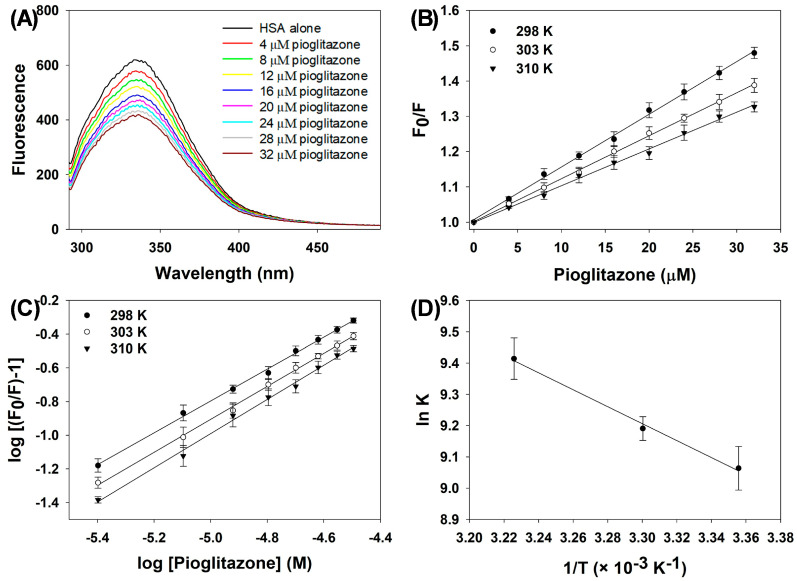
(**A**) Steady-state fluorescence emission spectra of HSA in the absence and presence of increasing concentrations of pioglitazone. (**B**) Stern–Volmer plots for the binding of pioglitazone to HSA at different temperatures. (**C**) Modified Stern–Volmer plots (double-log plots) for the binding of pioglitazone to HSA at different temperatures. (**D**) Van’t Hoff plots for the binding of pioglitazone to HSA at different temperatures. The assays were performed in triplicate, and the data is presented as mean values, with standard deviations indicated by error bars.

Fluorescence quenching spectroscopy is a commonly used method for investigating interactions between drugs and proteins. In this method, decreases in the fluorescence signal of a protein are analyzed to derive thermodynamic and binding parameters. Here, the fluorescence emission signal of HSA was examined in its free form and in complexes with varying concentrations of pioglitazone. HSA alone exhibits characteristic fluorescence with a maximum emission wavelength (λ_max_) at 335 nm. However, upon titration with pioglitazone, the fluorescence of HSA was quenched (Figure 3A). This alteration in fluorescence behavior indicates the formation of an HSA–pioglitazone complex. To analyze the fluorescence quenching data, the Stern–Volmer Equation (3) was employed:

(3) $$\frac{F_{0}}{F}=1+K_{SV}\left[Q\right]$$

where F_0_ represents the λ_max_ of HSA; F corresponds to the λ_max_ of the HSA–pioglitazone complex; K_SV_ denotes the Stern–Volmer constant; and [Q] represents the pioglitazone concentration. Additionally, the Stern–Volmer plot illustrating the quenching of HSA fluorescence by pioglitazone is depicted in Figure 3B. The fluorescence quenching assay was conducted at different temperatures (298, 303, and 310 K), yielding K_SV_ values of 1.525 ± 0.031 × 10^4^ M^−1^, 1.225 ± 0.063 × 10^4^ M^−1^, and 1.038 ± 0.068 × 10^4^ M^−1^, respectively (Table 2). Notably, two primary quenching modes were observed: static quenching and dynamic quenching. To gain further insights into the quenching mode, the bimolecular quenching rate constant (K_q_) was computed using Equation (4):

(4) $$K_{q}=\frac{K_{sv}}{\tau_{0}}$$

where τ_0_ is the integral fluorescence lifetime, with an average value of approximately 5.78 × 10^−9^ s. Additionally, K_q_ was determined at different temperatures: 298, 303, and 310 K, resulting in values of 2.640 ± 0.054 × 10^12^ M^−1^ s^−1^, 2.120 ± 0.110 × 10^12^ M^−1^ s^−1^, and 1.800 ± 0.119 × 10^12^ M^−1^ s^−1^, respectively (Table 2). Interestingly, when examining various quenchers of biological polymers, K_q_ values are generally reported to be approximately 2 × 10^10^ M^−1^ s^−1^ [24]. Notably, all tested temperatures yielded K_q_ values exceeding the maximum quenching rate constant typically observed for biopolymers, indicating a static mode of fluorescence quenching. To further confirm the quenching mode, the temperature dependence of K_SV_ was analyzed. In dynamic quenching, K_SV_ typically increases with rising temperature, whereas in static quenching, this trend is reversed. Our data revealed that K_SV_ values decreased as the temperature increased during the interaction between pioglitazone and HSA, reinforcing the conclusion that the quenching mode was static in nature.

The fluorescence quenching data was utilized to determine both the binding constant and the number of binding sites. To calculate the binding parameters, the modified Stern–Volmer equation (Equation (5)) was used:

(5) log F0−FF=lok Ka+n logQ

where n and K_a_ represent the binding sites and association constant, respectively. The modified Stern–Volmer plot (Figure 3C) revealed binding constants at temperatures of 298, 303, and 310 K as 0.863 ± 0.058 × 10^4^ M^−1^, 0.980 ± 0.037 × 10^4^ M^−1^, and 1.225 ± 0.079 × 10^4^ M^−1^, respectively (Table 2). These K_a_ values indicate moderate binding strength. Notably, the number of binding sites remained consistent across all temperatures, being approximately equal to one.

The binding constant (K_a_) was further utilized for the calculation of thermodynamic parameters. Primarily, non-covalent forces are involved in the complexation of drugs with proteins. Such interactions mainly encompass Van der Waals forces, H-bonds, electrostatic attractions, and hydrophobic forces [25]. The changes in entropy (ΔS°) and enthalpy (ΔH°) were determined using the van’t Hoff equation (Equation (6)):

(6) $$\ln K_{a}=-\frac{\Delta H{}^{\circ}}{RT}+\frac{\Delta S{}^{\circ}}{R}$$

where T and R refer to temperature and the gas constant (1.987 cal^−1^ mol K^−1^), respectively. Figure 3D illustrates the van’t Hoff plot depicting the interaction between pioglitazone and HSA. The thermodynamic parameters derived from this analysis are listed in Table 3. The change in Gibbs free energy (ΔG°) was computed using Equation (7):

(7) ΔG°=ΔH°−TΔS°

The binding of pioglitazone to HSA was spontaneous, as indicated by the negative Gibbs free energy values (ΔG° ranging from −5.361 ± 0.017 to −5.794 ± 0.022 kcal mol^−1^) [26]. The positive enthalpy change (ΔH° = 5.396 ± 0.220 kcal mol^−1^) suggests that hydrogen bonding contributes to the interaction [27]. In addition, the positive entropy (ΔS° = 36.099 ± 0.761 kcal mol^−1^ K^−1^) indicates that hydrophobic forces play a significant role, making the overall process entropically favorable. Thus, the complexation is driven by both enthalpic contributions (hydrogen bonds) and entropic contributions (hydrophobic interactions), consistent with the observed spontaneity of binding.

#### 2.2.2. Binding-Site Determination

Structural analyses confirm that HSA consists of three homologous domains—designated as domain I, domain II, and domain III. These domains are further subdivided into two distinct sub-domains, denoted as A and B. The precise binding sites for ligands within HSA are determined using site-specific probes with well-established binding preferences. In this investigation, two such markers were employed: warfarin and ibuprofen. Specifically, warfarin serves as a probe for sub-domain IIA (Sudlow’s site I), while ibuprofen binds to sub-domain IIIA (Sudlow’s site II) of HSA [28].

**Figure 4:**
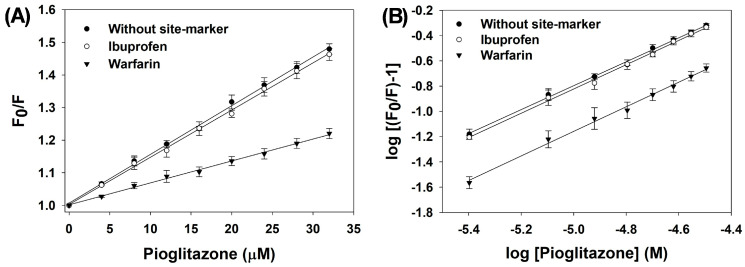
(**A**) Stern–Volmer plots for the competitive binding of pioglitazone with HSA in the absence and presence of site markers (ibuprofen or warfarin). (**B**) Modified Stern–Volmer plots for the competitive binding of pioglitazone with HSA in the absence and presence of site markers (ibuprofen or warfarin). The assays were performed in triplicate, and the data is presented as mean values, with standard deviations indicated by error bars.

A detailed analysis of the site-marker data was performed with the Stern–Volmer Equation (3). The Stern–Volmer plot for this assay is displayed in Figure 4A, while the corresponding K_SV_ values are provided in Table 4. Data show that K_SV_ values remained nearly constant when ibuprofen (1.460 ± 0.065 × 10^4^ M^−1^) was present compared to the absence (1.525 ± 0.031 × 10^4^ M^−1^) of a site marker, indicating that pioglitazone does not interfere with ibuprofen’s binding site on HSA. However, a noticeable decrease in K_SV_ value was observed in the presence of warfarin (0.680 ± 0.062 × 10^4^ M^−1^), suggesting that pioglitazone competes for the same binding region as warfarin, specifically sub-domain IIA. This observation supports the conclusion that pioglitazone binds primarily to sub-domain IIA of HSA.

Moreover, the values of K for the site-marker data were determined using Equation (5), and the corresponding plot is illustrated in Figure 4B. The K values are presented in Table 4. The K value without a site marker was 8.634 ± 0.589 × 10^3^ M^−1^. The binding constant for pioglitazone’s interaction with HSA showed minimal change in the presence of ibuprofen (9.540 ± 1.190 × 10^3^ M^−1^) but decreased significantly in the presence of warfarin (5.090 ± 0.845 × 10^3^ M^−1^). This further corroborates that pioglitazone binds to the same region on HSA as warfarin, sub-domain IIA. Certain HSA residues, particularly those located at 278–294 and 196–209, are highly susceptible to glycation. Specifically, within sub-domain IIA, residues such as Lys233, Lys199, Lys276, Lys525, Lys281, and Arg197 are prone to glycation [29]. Pioglitazone binding may reduce the exposure of glycation-prone amino groups, which could contribute to reduced glycation.

### 2.3. In Silico Analysis of HSA–Pioglitazone Complex and Its Dynamics

Molecular docking studies were conducted to evaluate the binding affinity between pioglitazone and HSA. Following this, the pioglitazone–HSA complex was subjected to molecular dynamics simulation to analyze its stability and dynamic behavior. The findings are discussed below.

#### 2.3.1. Molecular Docking

**Figure 5:**
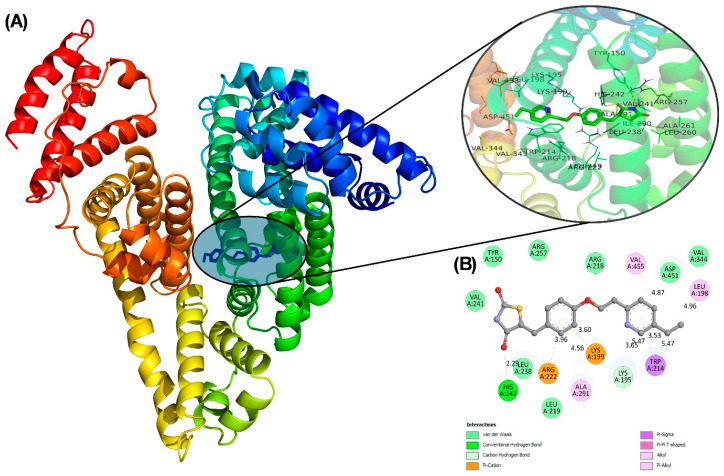
(**A**) Docked pose of pioglitazone bound to HSA. Enlarged view of interacting residues (circled). (**B**) Two-dimensional representation of the interacting residues of HSA with pioglitazone.

Bioinformatic tools like molecular docking and simulations are increasingly important in the study of drug–protein interactions, providing detailed insights into molecular mechanisms. These tools offer a high degree of accuracy, often generating results that mirror experimental data, making them invaluable in the early stages of therapeutic drug development [30]. For docking, pioglitazone was treated as a flexible ligand to determine its most stable conformations. The conformation with the lowest binding energy, −8.1 kcal mol^−1^, was selected for further analysis. Pioglitazone was found to interact with sub-domain IIA of HSA (Figure 5A). The docking results revealed that pioglitazone forms two hydrogen bonds with Lys195 and His242 in HSA at distances of 3.65 and 2.29 Å (Figure 5B). Additionally, other residues, including Ala291, Trp214, Leu198, and Val455, participated in hydrophobic interactions, while Arg222 and Lys199 were involved in ionic interactions with the drug. Further stabilization of the complex was provided by van der Waals interactions contributed by residues such as Leu238, Leu219, Val344, Asp451, Arg218, Arg257, Tyr150, and Val241. Pioglitazone was shown to bind within subdomain IIA of HSA, which is consistent with the experimental data. The interaction of pioglitazone with lysine and arginine residues of HSA highlights its role in preventing glycation, as these residues are known to undergo glycation-mediated modification. This is significant because glycation can impair protein function. Interestingly, capsaicin has also been found to interact with lysine and arginine residues in HSA [31], potentially preventing glucose from accessing these glycation-prone sites. By blocking these sites, pioglitazone may reduce the extent of glycation, offering a protective effect.

#### 2.3.2. Examination of Dynamics and Stability of the Complex Using MD Simulations

**Figure 6:**
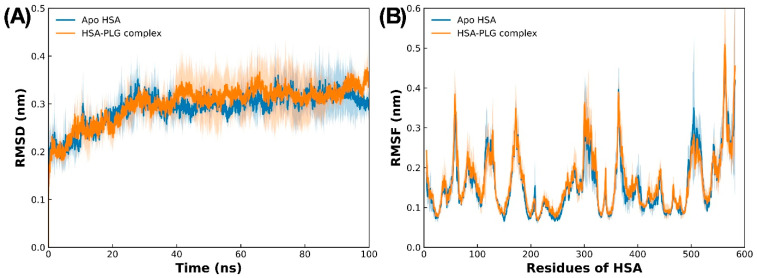
(**A**) Root mean square deviation of apo HSA and the HSA–pioglitazone complex during 100 ns MD simulations. (**B**) Average root mean square fluctuation (RMSF) of HSA residues in the absence and presence of pioglitazone. Data are presented as averages of three simulations, with SDs shown as shad regions.

The lowest binding energy pose obtained in the molecular docking study was used as the starting point in MD simulations to explore the dynamic behavior of the HSA–pioglitazone complex. To assess the stability of both apo HSA and the HSA–pioglitazone complex, the RMSD of backbone atoms was calculated (Figure 6A). The results showed that the system reached equilibrium at around 40 nanoseconds of simulation time, after which it remained stable. The RMSD of the HSA–pioglitazone complex closely followed that of apo HSA, suggesting that pioglitazone binding does not significantly affect the overall structural deviations of HSA during the simulation. The average RMSD values for apo HSA and the HSA–pioglitazone complex were 0.291 ± 0.031 and 0.298 ± 0.038 nm, respectively, further confirming that the HSA–pioglitazone complex maintained a stable trajectory in an aqueous environment.

In addition to RMSD analysis, the RMSF values of C_α_ atoms were computed for both apo and complex systems to examine how pioglitazone binding affects the flexibility of HSA residues. RMSF provides insight into the flexibility of specific protein regions over the simulation period. The RMSF data, presented in Figure 6B, show that pioglitazone binding did not significantly alter the fluctuation of HSA residues. Most residues exhibited minimal fluctuations, with RMSF values below 0.2 nm, indicating that pioglitazone binding does not induce major changes in the structural flexibility of HSA. This observation suggests that HSA retains a stable conformation even in the presence of the drug.

**Figure 7:**
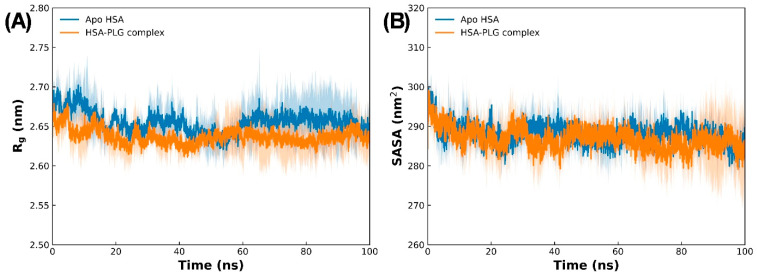
(**A**) Radius of gyration (R_g_) of apo HSA and HSA–pioglitazone complex during 100 ns MD simulations. (**B**) Solvent-accessible surface area of apo HSA and the HSA–pioglitazone complex during 100 ns MD simulations. Data are presented as averages of three simulations, with SDs shown as shad regions.

To further assess the stability of both apo HSA and the HSA–pioglitazone complex, additional parameters such as SASA and R_g_ were calculated. The radius of gyration is a critical measure in molecular dynamics simulations for evaluating the structural compactness and stability of proteins. In compact proteins, slight fluctuations in R_g_ are typically observed throughout the simulation, whereas more significant variations are common in expanded or unfolded proteins. This characteristic makes R_g_ a valuable metric for assessing the overall structural integrity and compactness of proteins in simulation studies. The results showed that the R_g_ of the HSA–pioglitazone complex was very similar to that of apo HSA (Figure 7A), indicating that pioglitazone binding did not cause structural changes in HSA. The average R_g_ for apo HSA was 2.655 ± 0.024 nm, while that of the HSA–pioglitazone complex was 2.637 ± 0.020 nm. These consistent R_g_ values suggest that both the apo HSA and the complex maintained structural compactness and stability throughout the simulation.

SASA was also calculated to further validate the stability of the protein–ligand complexes. SASA provides insight into the exposure of protein surfaces to solvent molecules, offering another means of assessing structural changes during simulations. The SASA values of HSA, both in the absence and presence of pioglitazone, are presented in Figure 7B. Similar to the findings with R_g_, the SASA of the HSA–pioglitazone complex was comparable to that of apo HSA throughout the simulation, further supporting the stability of the complex. The average SASA values for apo HSA and the HSA–pioglitazone complex were 288.126 ± 4.935 and 286.868 ± 3.823 nm^2^, respectively. These small differences, along with the consistency in SASA values during the simulation, indicate that the complex did not undergo significant structural changes once equilibrium was reached.

**Figure 8:**
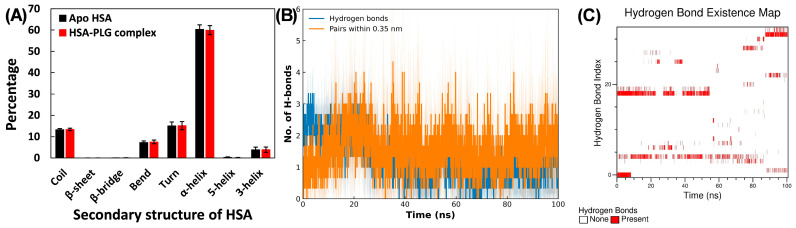
(**A**) Average percentages of secondary structural elements in HSA in the absence and presence of pioglitazone. (**B**) Number of hydrogen bonds formed between HSA and pioglitazone during 100 ns MD simulations. (**C**) Hydrogen bond existence map showing hydrogen bond formation between HSA and pioglitazone during 100 ns MD simulations. Data are presented as averages of three simulations, with SDs shown as shaded regions.

The impact of pioglitazone binding on the structural stability of HSA was examined by analyzing the secondary structure components of HSA. The secondary structure compositions of apo HSA and the HSA–pioglitazone complex are displayed in Figure 8A. In the case of apo HSA, the α-helical content was found to be 60.310 ± 2.103%, a value consistent with reports in the literature. When pioglitazone was bound to HSA, the α-helical content remained nearly unchanged, slightly decreasing to 59.925 ± 2.128%. In addition to α-helical content, other secondary structure elements such as coils, bends, and turns were analyzed. In apo HSA, the average percentages of these components were 13.264% for coils, 7.266% for bends, and 15.086% for turns. These structural elements remained nearly unchanged upon pioglitazone binding, further reinforcing the notion that the secondary structure of HSA was preserved. The preservation of α-helical content suggests that pioglitazone binds without perturbing the overall fold of HSA and may therefore reduce the accessibility of glycation-prone side chains through local shielding rather than inducing large conformational rearrangements.

The interaction between pioglitazone and HSA was further analyzed by examining the hydrogen bond profile (Figure 8B). On average, 1.031 hydrogen bonds were formed between pioglitazone and HSA throughout the simulation. The data revealed that hydrogen bonds were consistently present across nearly all frames of the simulation trajectory. To provide a more detailed understanding of these interactions, a hydrogen bond existence map was created (Figure 8C), which confirmed the continuous presence of H-bonds throughout the simulation period. Additionally, H-bond occupancy was also calculated. The residues Arg257 and Lys199 exhibited the highest H-bond occupancies of 35.7% and 32.8%, respectively. Other hydrogen bonds were also observed during the trajectory, but with lower occupancies. It is worth noting that lysine and arginine residues showed the highest hydrogen bond occupancy. As these residues are highly prone to glycation, binding of pioglitazone to these sites on HSA could potentially reduce glucose accessibility, ultimately inhibiting glycation.

**Figure 9:**
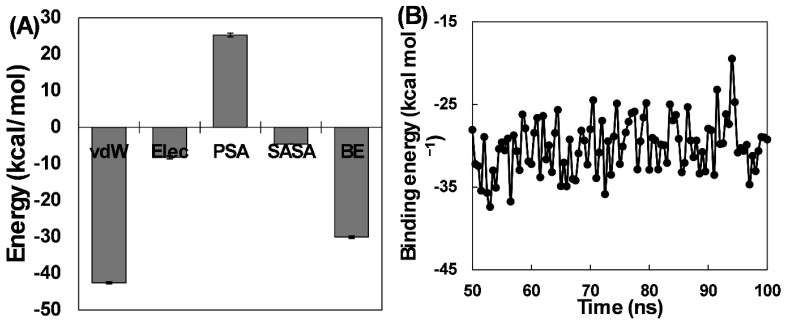
(**A**) Individual components of the binding energies for the interaction between pioglitazone and HSA. MM-PBSA energies were calculated from 100 frames spanning 50–100 ns. (**B**) Frame-wise overall binding energies for the 100 frames spanning 50–100 ns.

The interaction forces between pioglitazone and HSA were examined using MM-PBSA analysis. Binding energies were determined by sampling 100 frames from the 50–100 ns segment of the trajectory, and the data is shown in Figure 9A. In drug–protein interactions, the primary non-covalent forces include H-bonds, hydrophobic forces, electrostatic interactions, and Van der Waals interactions, which can either support or hinder overall binding [32]. Van der Waals interactions were the most significant contributors to the binding of pioglitazone with HSA, with an energy of −42.52 ± 0.28 kcal mol^−1^, followed by electrostatic interactions at −8.22 ± 0.41 kcal mol^−1^. Additionally, SASA energy contributed −4.57 ± 0.02 kcal mol^−1^ to the overall binding energy. Conversely, polar solvation energy, with a value of 25.26 ± 0.53 kcal mol^−1^, impeded the interaction, as indicated by its positive value. The total binding energy for pioglitazone–HSA interaction was calculated to be −30.06 ± 0.31 kcal mol^−1^. To ensure the validity of MM-PBSA calculations, the binding energy of each frame was assessed, and all frames exhibited negative binding energies, supporting the average binding energy value (Figure 9B).

The MM-PBSA data also provide the energy contributions of individual residues. Table 5 shows the energy contributions of residues with the highest binding energy contributions. Trp214 showed highest energy contribution at −2.66 ± 0.04 kcal mol^−1^, followed by Lys195 at −2.11 ± 0.09 kcal mol^−1^, and Arg218 at −1.93 ± 0.07 kcal mol^−1^. Notably, many of the top energy-contributing residues were lysine and arginine. Since glycation predominantly occurs at these residues, pioglitazone binding to these glycation-prone sites on HSA could potentially inhibit glycation.

## 3. Materials and Methods

### 3.1. Stock Solution Preparation

HSA (A3782), pioglitazone (CDS021593), warfarin (45706), and ibuprofen (PHR1004) were obtained from Sigma-Aldrich, Saudi Arabia. The glycation assays were carried out using a sterile phosphate buffer (50 mM Na phosphate with pH 7.4). The HSA stock solution was prepared in this buffer at a 600 µM concentration. Stock solutions of pioglitazone, warfarin, and ibuprofen were prepared separately in dimethyl sulfoxide (DMSO) and subsequently diluted in the same buffer. Throughout the experiment, the final DMSO concentration was below 0.5% *v*/*v* to minimize any potential solvent interference.

### 3.2. Glycation Assays

#### 3.2.1. Experimental Setup for HSA Glycation

All glycation assays were performed in vitro. HSA glycation was induced with glucose as the glycating agent, following a standard protocol with minor changes [33]. A mixture of 30 mM glucose and 300 µM HSA was incubated in the treated group, along with varying concentrations (50–200 µM) of pioglitazone. D-Glucose was used to induce glycation because it is the predominant reducing sugar in plasma and best models chronic hyperglycaemia in vivo. The native HSA group contained only HSA, without any added glucose. The glycated HSA group comprised both HSA (300 µM) and glucose (30 mM). To prevent microbial contamination, 0.02% (w/v) sodium azide was included in all samples. Treatment with 5 mM aminoguanidine was given in the positive control. Aminoguanidine was included for antiglycation because it is a well-established inhibitor of AGE formation in vitro, acting primarily by trapping reactive dicarbonyl intermediates. The concentration (5 mM) was chosen based on prior in vitro studies demonstrating robust inhibition under accelerated glycation conditions [34]. The samples were then incubated for four weeks at 37 °C. After incubation, the samples were dialyzed against phosphate buffer. Subsequently, protein concentration was examined using the Lowry assay [35], and sample volumes were adjusted to ensure equal HSA concentrations across all samples. Finally, the samples were aliquoted and stored at −20 °C for subsequent analysis.

#### 3.2.2. Estimation of Fructosamine Content

Fructosamine levels in various HSA samples were quantified using a modified NBT (nitro blue tetrazolium) method, as described earlier [36]. First, the HSA samples were diluted in 100 mM Na_2_CO_3_ buffer with a pH of 10.8. The diluted samples were then mixed with NBT (1.0 mM) and incubated for 2 h at 37 °C. Following incubation, absorbance was measured at 530 nm. Fructosamine content was quantified in nanomoles per milligram of protein, using ε = 12,640 cm^−1^ mol^−1^ [37].

#### 3.2.3. Determination of Fluorescent AGEs

Samples were diluted to 10 µM to detect fluorescent AGEs. Subsequently, fluorescence intensity was measured using a spectrofluorometer with λ_ex_ = 370 nm. Emission was recorded at a wavelength of 440 nm, following standard methodology [38]. The fluorescence signal at λ_em_ = 440 nm served as the basis for calculating the percentage of AGE inhibition.

#### 3.2.4. Free Lysine Modification

In this study, modifications of free lysine residues in HSA were investigated in all samples using the TNBSA reagent, following established protocols [39]. To begin, protein samples were diluted in NaHCO_3_ buffer (pH 8.5, 100 mM). For each sample, 500 μL of diluted protein was mixed with 0.25 mL of 0.01% TNBSA solution, followed by 2 h of incubation at 37 °C. After this, 250 μL of SDS (10%) solution and 0.125 mL of 1 N HCl were added to each sample. Finally, the optical density at 335 nm was measured using a spectrophotometer.

#### 3.2.5. Carbonyl Content Assessment

To assess lysine content, the Levine method was employed with minor changes [40]. Briefly, samples were diluted in phosphate buffer, and 0.6 mL of the diluted samples was added to 0.4 mL of pre-chilled 40% TCA (trichloroacetic acid). After centrifugation, the resulting pellet was reconstituted in 0.4 mL PO_4_-buffered saline. Subsequently, 0.4 mL DNPH (2,4-dinitrophenylhydrazine), prepared in 4 N HCl at a concentration of 20 mM, was added. Incubation was carried out at 37 °C for 90 min. Following incubation, 0.35 mL of TCA was added, and samples were centrifuged again. The supernatant was discarded, and the protein pellet was solubilized in 6 M guanidine HCl. Absorbance was measured at 360 nm, and carbonyl content was quantified in nanomoles per mg of protein using ε = 22,000 M^−1^ cm^−1^.

#### 3.2.6. Circular Dichroism (CD) Measurements

CD spectra of all HSA samples were recorded using a spectropolarimeter at 37 °C. Samples were diluted to 5 µM in Na phosphate buffer, and spectra were recorded in the 195–260 nm range using a 1 mm path-length quartz cuvette. Subsequently, α-helices in HSA samples were computed using standard equations.

### 3.3. In Vitro Interaction of Pioglitazone with HSA

In this study, spectrofluorometric measurements were performed to record the fluorescence spectrum of HSA using a spectrofluorometer. Briefly, HSA was excited at 280 nm, and the emission spectrum was recorded in the 290–500 nm range. Following this, increasing concentrations of pioglitazone were added to the HSA solution, and the corresponding fluorescence emission was measured at each titration point.

In the site-marker displacement assays, HSA at a concentration of 5 µM was incubated with 10 µM of site markers—either ibuprofen or warfarin—to saturate the HSA. The fluorescence of the site-marker-saturated HSA was recorded upon excitation at 280 nm. Saturation was performed to ensure that all binding sites in HSA were occupied by the site markers. Subsequently, increasing concentrations of pioglitazone were introduced, and the resulting fluorescence signals were measured. Each site-marker experiment was conducted independently to assess the displacement effect of pioglitazone.

### 3.4. In Silico Studies

Molecular docking of pioglitazone with HSA was performed to examine binding affinity. The resulting complex was then subjected to molecular dynamics simulation to assess stability and dynamics. The detailed procedure is described below.

#### 3.4.1. Molecular Docking

Molecular docking studies were performed to investigate the interaction between pioglitazone and HSA using AutoDock Vina [41]. The 3D structure of HSA was retrieved from the RCSB Protein Data Bank (ID: 1AO6). Prior to docking, crystal water molecules were removed and non-polar hydrogens were added. Using AutoDockTools-1.5.6, Kollman charges were assigned. To encompass the entire HSA structure, grid spacing was set to 1 Å. The structure of pioglitazone was sourced from PubChem [CID: 60560], and the ligand was allowed to adopt a flexible conformation to identify the binding mode with the lowest binding energy. Finally, the resulting conformation was analyzed using PyMOL-3.1.3.1 and Discovery Studio.

#### 3.4.2. Molecular Dynamics Simulations

To initiate MD simulations, the lowest-energy HSA–pioglitazone complex obtained through docking was used. Molecular simulations were carried out using Gromacs-2018.1 with the Amber99sb-ildn force field [42,43]. To prepare the system, the pioglitazone topology was generated using the Antechamber package [44] within AmberTools, and atomic charges were assigned using the AM1-BCC charge model. Both apo HSA and the complex were solvated in a cuboidal box and neutralized by adding 15 sodium (Na) atoms. To mimic physiological conditions, 150 mM NaCl was added to the system. Energy minimization was performed using the steepest descent minimization algorithm, with a cut-off radius of 12 Å for non-bonded interactions, to eliminate weak Van der Waal contacts. Subsequently, equilibration was performed under the NVT ensemble using a V-rescale thermostat at 310 K for 1000 ps, followed by the NPT ensemble at 1.0 bar for an additional 1 ns using the Parrinello–Rahman barostat [45,46]. Standard MD simulations were run for 100 ns using the LINCS algorithm. Three independent replicates of MD simulations were performed, and the results are presented as averages across replicates. Prior to subsequent analysis, PBC corrections were applied to trajectories. Structural properties, including root mean square fluctuation (RMSF), root mean square deviation (RMSD), solvent-accessible surface area (SASA), secondary structure content, radius of gyration, and hydrogen bonds, were analyzed using Gromacs utilities. Furthermore, MM-PBSA analysis was performed to estimate binding energies associated with the formation of the HSA–pioglitazone complex [47].

### 3.5. Statistical Analysis

In vitro glycation assays were conducted in quadruplicate, and the results are presented as mean values with standard deviations. Binding assays were performed in triplicate, and the results are presented as mean values with standard deviations. The *p*-value was calculated using one-way ANOVA followed by appropriate post hoc tests. A *p*-value ≤ 0.05 was considered statistically significant. Molecular simulations were performed in triplicate, and the results are presented as mean values with standard deviations.

## 4. Conclusions

This study elucidates the antiglycation potential of pioglitazone, demonstrating its ability to inhibit over 70% of glycation formation in HSA. The findings indicate that pioglitazone not only reduces harmful modifications associated with glycation but also improves biochemical markers. Spectroscopic and docking studies suggest that pioglitazone binding at Sudlow’s site IIA may reduce the accessibility of glycation-prone residues. These findings suggest that pioglitazone has substantial antiglycation potential, offering a promising strategy for mitigating diabetic complications by targeting glycation processes. Pioglitazone exhibits antiglycation activity in vitro and preserves several biochemical markers of HSA integrity. If validated in cellular and in vivo models, the observed antiglycation effects of pioglitazone could support its potential use in the management of diabetic complications. Given the central role of HSA in plasma redox balance and ligand transport, these findings warrant further investigation in cellular and in vivo models to determine whether pioglitazone can meaningfully reduce glycoxidative stress and improve clinical outcomes associated with AGE-related pathology.

## Figures and Tables

**Table 1:** Molar ellipticity at 208 nm and percentages of α−helices in native HSA, glycated HSA, and pioglitazone-treated HSA.

Samples	MRE_208nm_	% α-helix
Native HSA	−20,897.343	58.26
Glycated HSA	−13,004.216	31.04
HSA with 200 µM pioglitazone	−18,216.247	49.02

**Table 2:** Values of the Stern–Volmer constants, quenching rate constants, binding constants, and numbers of binding sites for the interaction between pioglitazone and HSA at varying temperatures, obtained using the fluorescence quenching method.

Temp (K)	K_SV_ (×10^4^ M^−1^)	K_q_ (×10^12^ M^−1^ s^−1^)	K (×10^4^ M^−1^)	n
298	1.525 ± 0.031	2.640 ± 0.054	0.863 ± 0.058	0.980 ± 0.039
303	1.225 ± 0.063	2.120 ± 0.110	0.980 ± 0.037	0.979 ± 0.035
310	1.038 ± 0.068	1.800 ± 0.119	1.225 ± 0.079	0.981 ± 0.030

K_SV_ is the Stern–Volmer constant; K_q_ is the quenching rate constant; n is the number of binding sites; and K is the binding constant. The assays were performed in triplicate, and the data is presented as mean values with standard deviations.

**Table 3:** Values of the thermodynamic parameters for the binding of pioglitazone with HSA at varying temperatures, obtained using the steady-state fluorescence method.

Temp (K)	ΔG° (kcal mol^−1^)	ΔH° (kcal mol^−1^)	ΔS° (kcal mol^−1^ K^−1^)
298	−5.361 ± 0.017		36.099 ± 0.761
303	−5.541 ± 0.019	5.396 ± 0.220	
310	−5.794 ± 0.022		

ΔH°, ΔG°, and ΔS° are the changes in enthalpy, free energy, and entropy, respectively, and T is the temperature in Kelvin. The assays were performed in triplicate, and the data is presented as mean values with standard deviations.

**Table 4:** K_SV_ and K values for pioglitazone and HSA interaction without and with site markers.

Samples	K_SV_ (×10^4^ M^−1^)	K (×10^3^ M^−1^)
Without site marker	1.525 ± 0.031	8.634 ± 0.589
With ibuprofen	1.460 ± 0.065	9.540 ± 1.190
With warfarin	0.680 ± 0.062	5.090 ± 0.845

K_SV_ is the Stern–Volmer constant, and K is the binding constant. The assays were performed in triplicate, and the data is presented as mean values with standard deviations.

**Table 5:** Polar energy, apolar energy, and total energy of the highest energy-contributing HSA residues involved in the interaction with pioglitazone.

Residue	Polar Energy	Apolar Energy	Total Energy
Lys195	0.519 ± 0.226	−0.191 ± 0.005	−2.111 ± 0.096
Leu198	0.491 ± 0.017	−0.137 ± 0.003	−1.491 ± 0.033
Trp214	0.837 ± 0.040	−0.257 ± 0.004	−2.662 ± 0.045
Arg218	−1.277 ± 0.139	−0.071 ± 0.006	−1.934 ± 0.078
Arg222	−0.731 ± 0.033	0.000 ± 0.000	−0.812 ± 0.032
Leu347	−0.068 ± 0.005	−0.075 ± 0.002	−0.779 ± 0.026
Arg348	−1.293 ± 0.022	0.000 ± 0.000	−1.160 ± 0.023
Lys436	0.730 ± 0.168	−0.061 ± 0.005	−1.016 ± 0.069
Tyr452	0.391 ± 0.024	−0.112 ± 0.003	−1.497 ± 0.043
Val455	−0.320 ± 0.009	−0.068 ± 0.002	−1.279 ± 0.027
Arg484	−1.246 ± 0.032	0.000 ± 0.000	−1.143 ± 0.028
Arg485	−0.972 ± 0.015	0.000 ± 0.000	−0.933 ± 0.015

## Data Availability

The data is available from the corresponding author on request.
